# Spatial Skills Associated With Block-Building Complexity in Preschoolers

**DOI:** 10.3389/fpsyg.2020.563493

**Published:** 2020-10-22

**Authors:** Xiaoxia Zhang, Chuansheng Chen, Tao Yang, Xiaohui Xu

**Affiliations:** ^1^Collaborative Innovation Center of Assessment for Basic Education Quality, Beijing Normal University, Beijing, China; ^2^School of Education, Capital Normal University, Beijing, China; ^3^Department of Psychological Science, University of California, Irvine, Irvine, CA, United States; ^4^School of Preschool Education, Capital Normal University, Beijing, China

**Keywords:** block building complexity, form perception, spatial visualization, spatial skill, preschooler

## Abstract

Block building is a popular play activity among young children and is also used by psychologists to assess their intelligence. However, little research has attempted to systematically explore the cognitive bases of block-building ability. The current study (*N* = 66 Chinese preschoolers, 32 boys and 34 girls; mean age = 4.7 years, SD = 0.29, range = 3.4 to 5.2 years) investigated the relationships between six measures of spatial skills (shape naming, shape recognition, shape composition, solid figure naming, cube transformation, and mental rotation, with the former four representing form perception and the latter two representing visualization) and block-building complexity. Correlation results showed that three of the four measures of form perception (shape naming, shape recognition, and shape composition) were significantly and positively correlated with block-building complexity, whereas the two measures of visualization were not. Results from regression models indicated that shape recognition and shape composition, as well as shape-recognition-by-gender interaction, were unique predictors of children’s block-building complexity. These findings provide preliminary evidence for the basic spatial skills underlying children’s block-building complexity and have implications for classroom instructions aimed at improving preschoolers’ block-building complexity.

## Introduction

Block play is a popular activity amongst preschoolers (Varol and Farran, 2006; Schmitt et al., 2018) and has been deemed by researchers as a versatile activity to help children develop technological thinking, critical thinking, problem solving, creativity, and abstract thinking (Reifel, 1984; Robbins et al., 2011; Otsuka and Jay, 2017; Schmitt et al., 2018). Not surprisingly, psychologists have also used block building to measure children’s intellectual development (Caldera et al., 1999; Hayashi and Takeshita, 2009; Ness and Farenga, 2016). Empirical studies have further found that preschool children who showed a high level of block construction would attain better math and reading achievement during their school years (elementary through high school), even after controlling for other general cognitive abilities (Wolfgang et al., 2001, 2003; Hanline et al., 2010; Nath and Szücs, 2014; Richardson et al., 2014; Verdine et al., 2014b).

The aim of the current study was to search for specific spatial abilities that serve as basic cognitive foundations for block building in preschool children. We first describe the types of block-building activities and related measures of performance, then review the literature on factors that contribute to block-building ability with a specific emphasis on spatial abilities, and finally introduce the current study.

### Types of Block-Building Activities and Measures of Performance

There are three types of block-building activities: structured, unstructured (free block play), and semi-structured block building. In the structured block play, children were asked to duplicate the given models using blocks of various sizes and shapes (Caldera et al., 1999; Cohen and Emmons, 2017; Schmitt et al., 2018). Examples of structured block play include “Stacking blocks”, “Three-Dimensional Constructional Praxis” (Benton and Fogel, 1962; Hayashi and Takeshita, 2009), Legos, or Mega Blocks (TOSA). Children were asked to complete the task within a limited amount of time (Verdine et al., 2014a,b). Children’s performance is evaluated using two types of criteria: Match scoring and Dimensional scoring. Match scoring counts the number of blocks correctly placed (Benton and Fogel, 1962). Some researchers adopted the stringent criterion of scoring a point only if the child correctly stacked 100% of the blocks (Hayashi and Takeshita, 2009). Dimensional scoring takes into consideration the processes and mistakes in block building. Specifically, children’s performance was assessed in terms of two aspects: the overall accuracy of the whole product relative to the central piece and the complexity of multiple component pieces (Verdine et al., 2014b).

Unstructured block play is a self-initiated, self-guided, and open-ended play activity. In other words, children can build whatever they want without instructions. To assess children’s performance in free block building, researchers have coded children’s construction behaviors, such as sharing with others, pauses for reflection, and satisfaction for self-directed play (Otsuka and Jay, 2017), or coded the end products in terms of complexity and the number and variety of blocks used (Caldera et al., 1999). Other researchers have focused on the developmental progression of block building. For example, Reifel (1984) found that children went through the following sequence: stacking, row construction, combination of stacking and row construction, piling (three dimensions with no interior space), enclosure (flat), enclosure (arches), enclosure (combination), and finally combination of many forms. Later on, Hanline et al. (2001) condensed the sequence into five stages by focusing on spatial dimensionality change: non-construction, linear construction, bidimensional construction, tridimensional construction, and representational construction.

Between the two extremes of structured and free block play lies semi-structured block play, in which an adult, such as a teacher, provides a prompt at the beginning but then lets children work freely with minimum involvement from the adult. The prompt can be as specific as constructing a specific house as shown on a poster (Casey et al., 2008) or as general as building a school with four walls and at least two rooms (Ramani et al., 2014; Schmitt et al., 2018). The adult can also ask children to show the story they hear by using blocks (Reifel and Greenfield, 1983). Researchers have grappled with a variety of ways to assess children’s performance during semi-structured block building. Some researchers (Casey et al., 2012) have emphasized structural balance. Other researchers have focused on the number and type or even the symbolic meaning of structures. For example, Ramani et al. (2014) used four criteria: the combined number of blocks in height and in length, number of different columns and rows, meaningful use of the colors and shapes, and number of bridge formations. Still other researchers (Reifel, 1984; Hanline et al., 2001) have paid attention to developmental progression (as discussed earlier for free play). Finally, Casey et al. (2008) adapted an assessment tool developed for free block building to assess semi-structured construction. They added hierarchical integration to capture increasing structural complexity. Hierarchical integration occurs when children combine blocks to create more complex structures with vertical interior space, such as an arch or a bridge (Casey et al., 2008).

Although all three types of block-building activities have been used in the literature, semi-structured block building has several advantages over the other two when assessing children’s block-building ability. First, unlike structured block play, semi-structured block play allows children to use their spatial skills and creativity to complete the task in any multiple of ways they prefer (Reifel and Greenfield, 1983; Ramani et al., 2014). Second, semi-structured play overcomes the drawbacks of unstructured free play which typically leads to simple structures and constant changes in children’s building plans (Casey et al., 2008). Finally, semi-structured prompts can be easily adapted for use as an instructional strategy to enhance children’s learning during free choice time (Schmitt et al., 2018).

### Factors That Contribute to Children’s Block-Building Ability

Researchers have examined various factors related to children’s block-building ability. In terms of demographic factors, it is expected that children’s block-building level increases with their chronological age (Hanline et al., 2001). The evidence regarding gender, however, has been mixed. Some research showed no significant gender difference in the complexity of block building (Hanline et al., 2001; Verdine et al., 2014b), but girls tended to build more house features, such as walls, windows, and doors, than did boys (Ramani et al., 2014). Other researchers, however, revealed that boys outperformed girls in block-building skills in China (Tian et al., 2018).

In terms of cognitive factors, Tian et al. (2019) recently proposed a conceptual model that abstract reasoning, numeracy, representational thinking, and spatial ability are the underlying cognitive mechanisms for block play. Among these cognitive capacities, spatial ability is the most crucial (Tian et al., 2019). Spatial ability includes several subcomponents. Based on their meta-analysis, Linn and Petersen (1985) concluded that spatial ability included spatial perception, mental rotation, and spatial visualization. Of the three subcomponents, mental rotation and spatial visualization involve a shared cognitive skill of forming and manipulation mental image (Hawes et al., 2017). A later factor-analysis by Carroll (1993) identified five major spatial abilities including visualization, spatial relations, flexibility of closure, perceptual speed, and closure speed. Finally, a more recent meta-analysis grouped the spatial abilities into two categories: small-scale spatial abilities (including allocentric spatial transformation, such as mental rotation along the object’s central axis and object manipulation) and large-scale spatial abilities (including egocentric spatial transformation, such as environmental navigation and mental rotation along the body axis) (Wang et al., 2014). Most recently, Mix et al. (2016, 2017) reviewed a series of spatial tasks used to test subjects across a large age range from kindergarten to sixth grade and concluded that spatial ability included three dimensions: spatial visualization, form perception, and spatial scaling. Despite the variations across the above studies, two key subcomponents of spatial skills seem to be closely related to block building: spatial visualization and form perception. Spatial visualization is the ability to imagine and mentally manipulate figures or objects in space and it can be measured by mental rotation and perspective thinking tasks as well as through a particular type of block design task (using cubes with red and white sides to produce a 3-D structure according to a series of 2-D figure patterns). Form perception is the ability to recognize shapes, distinguish them from their backgrounds, and decompose them into parts, and this skill can be measured with tasks such as figure copying and visual spatial working memory (Mix et al., 2016, 2017).

Thus far, empirical evidence has been mixed in terms of the relationship between spatial visualization and block-building ability. In adult patients with cerebral disease, spatial visualization (perception of orientation or location) strongly predicted accuracy on the structured block play task (Capruso and Hamsher, 2011). Two studies of preschoolers, however, found no significant association between spatial visualization (assessed using Block Design) and block-building ability (Caldera et al., 1999; Casey et al., 2008). When spatial visualization was measured with a mental rotation task, a study of 9-year-old children found that 2-D mental rotation performance was significantly associated with block-building ability (Brosnan, 1998). Consistent with that finding, training with a structured block play game was found to improve 8-year-old children’s 2-D letter mental rotation ability with associated changes in brain activation (Newman et al., 2016). However, another study that trained 5.6- to 6.7-year-old children on block building (with semi-structured storytelling block building or imitation of poster block building) did not lead to improvement in 3-D mental rotation (Casey et al., 2008). It seems possible that both age of the participants (older but not younger ones showed significant associations between visualization and block building) and the task (3-D mental rotation may be too difficult for young children (Levine et al., 2012) affect the outcome.

In contrast to the handful of studies on the association between spatial visualization and block building, little is known about the relationship between form perception and block-building play. Thus far, only two relevant studies have been conducted. Caldera et al. (1999) found that block-building ability was significantly associated with geometric figure abstraction ability [which is similar to the subcomponent of Mix et al. (2016, 2017) of form perception]. More recently, an intervention study showed that 7 weeks of semi-structured block intervention resulted in improved shape recognition for children aged from 38 to 69 months (Schmitt et al., 2018). Thus far, no systematic research has been conducted using various measures of form perception.

### The Current Study

**FIGURE 1:**
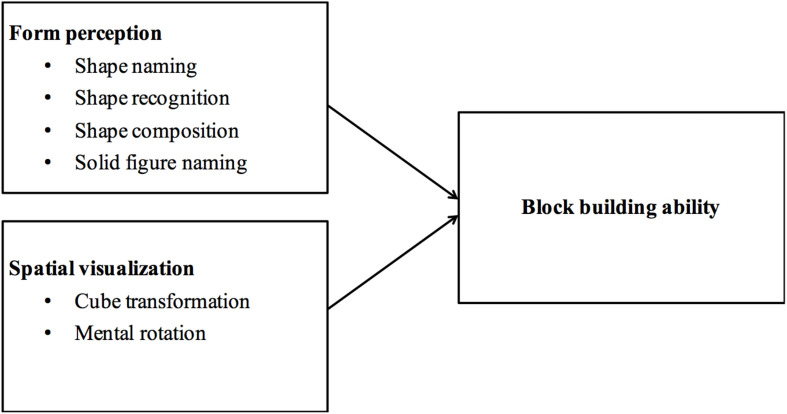
Schematic model of spatial skills underlying block-buildings ability.

To expand the limited literature on the cognitive bases of block building, this study examined the associations between multiple measures of form perception and spatial visualization and preschoolers’ block-building ability (see Figure 1). Four measures of form perception were used: 2-D shape naming, shape recognition, shape composition, and 3-D solid figure naming. This selection of measures was based on the standard conceptualization of form perception in geometry for early education (Sarama and Clements, 2009; Common Core State Standards Initiative, 2010; Sinclair and Bruce, 2015), according to which, form perception includes shape naming, 2-D and 3-D solid shape identification, and shape composition. For spatial visualization, we used two measures: 2-D mental rotation (because 3-D might have been too difficult for preschoolers, as discussed earlier) and cube transformation (2D-3D spatial transformation).

Block-building ability was assessed using semi-structured block construction because of its advantages over structured and free block play, as mentioned earlier. The index for block-building ability used the same complexity as used in previous studies (Hanline et al., 2001; Stannard et al., 2001).

Our main analyses focused on the relations between the six measures of spatial skills and block-building ability. We examined their bivariate relations (via Pearson product-moment correlations) as well as unique contributions (via multiple regression analysis). Finally, given the possible role of gender in spatial ability and block-building ability (as discussed earlier), we also explored potential interactions with gender.

## Materials and Methods

### Participants

Participants were 66 second-year preschoolers (32 boys and 34 girls) from a public preschool in a middle-class neighborhood in Beijing. Children ranged in age from 3.4 to 5.2 years (M_age_ = 4.7 years, SD = 0.29). The protocol of this study was approved by the IRB of the Collaborative Innovation Center of Assessment for Basic Education Quality, Beijing Normal University. Parental consent was obtained for each child before the experiment. Children were allowed to terminate the experiment at any point during the experiment. No child made such a request. This study was based on the data from another study (Zhang et al., 2014).

### Materials

#### Block Building Test

**FIGURE 2:**
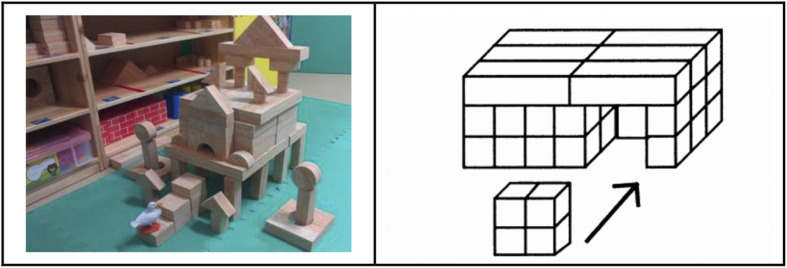
An example of a finished product scored as 9 (on the left panel) and the defining characteristics of products scored as 9 (3-d horizontal closure structure with 2 block-high, roof and internal space) (on the right panel).

Casey’s *Block Building Measure* (Casey et al., 2008) was used to assess children’s block-building ability. Children were provided with 70 different shapes of unit blocks and asked to build a house with a ceiling that prevents raindrops from reaching the inside when it rains outside. Children were allotted 12 min to finish the house. This test has shown good reliability and validity (Casey et al., 2008). Children’s block constructions were coded into 14 levels with scores ranging from 0 to 8.5: random placement (0), 1-d structure (1), 2-d with no internal space (2), 2-d with vertical internal space (3), 2-d with horizontal internal space (4), 2-d with horizontal internal space and no gaps (4.5), 3-d structure without internal space (5), 3-d structures with internal space and depth (6), series of arches (6.5), 3-d structure with irregular 1 block-high enclosure and roof (7), 3-d structure with regular 1 block-high enclosure and roof (7.5), 3-d structure with irregular 2-block high enclosure (8), 3-d structure with regular 2-block high enclosure (8.5), and 3-d horizontal closure structure with 2 block-high, roof and internal space (9) (Casey et al., 2008). Figure 2 presents an example of the finished product scored as 9.

#### Form Perception Measures

Four tasks were used to measure form perception: shape naming, shape recognition, shape composition, and solid figure naming. The four tasks were scored separately. Because the four tasks had different ranges of scores, they were first standardized and then averaged to create an index of form perception. Reliability for the form perception subscores was Cronbach’s α = 0.82.

During the shape naming task, children were asked to say the name after being shown a series of ten shapes: square, parallelogram, trapezoid, semi-circle, pentagram, oval, and sector. Children received 1 point for each correctly named shape. The total score on this task could range from 0 to 7. This test was developed by Clements et al. (1999), who did not report reliability. It was also used by Verdine et al. (2016) to assess children’s knowledge of shape names and they did not report reliability either. Cronbach’s α in the current study was 0.60.

**FIGURE 3:**
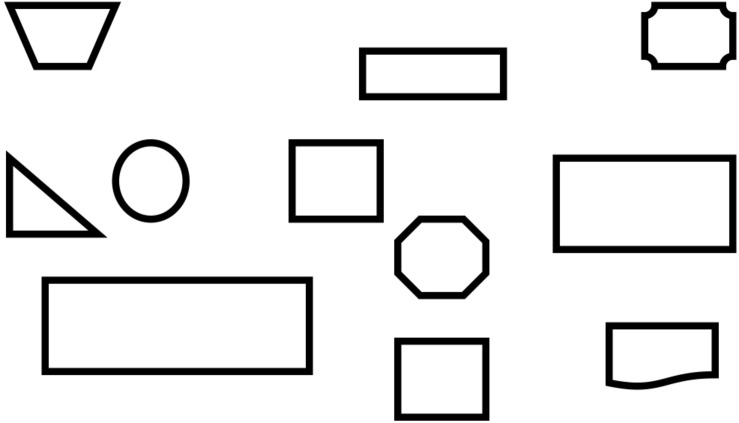
A sample item of rectangle recognition from the shape recognition task.

The shape recognition task was based on previous research (Clements et al., 1999; Schmitt et al., 2018). There are 10 picture boards, with each containing three target shapes (e.g., three rectangles) and a number of distractor shapes (e.g., triangles, parallelograms, and irregular shapes) (see Figure 3). Children were asked to point out all target shapes on each board. They were given 1 point for recognizing all three target shapes for each trial. The total score on this task could range from 0 to 10. This task has shown good reliability (0.73) in a previous study (Schmitt et al., 2018), although Cronbach’s α in the current study was a little lower, at 0.57.

During the shape composition task, children were asked to compose new shapes of their choice from triangles that were provided to them. Children were first given two right triangles to compose a new shape, and then subsequently offered four right triangles to compose a new shape. After they finished each composition, children were asked to name the new shape. Two right triangles can be combined to form a new large triangle, a rectangle, or a parallelogram. Four right triangles can be combined to form a large triangle, a rectangle, a parallelogram, a trapezoid, or a square. Children received 1 point for correctly composing a shape and 1 point for correctly naming it. The total score on this task could range from 0 to 16. This test was developed by authors of the current study. Cronbach’s α in the current study was 0.71.

In the solid figure naming task, children were presented with a cube, a cuboid, a cylinder, and a triangular prism and were asked to name these 3-D figures. Children received 2 points for correctly naming each figure. The total score on this task could range from 0 to 8. This test was developed by authors of the current study. Cronbach’s α in the current study was 0.78.

#### Spatial Visualization Measures

**FIGURE 4:**
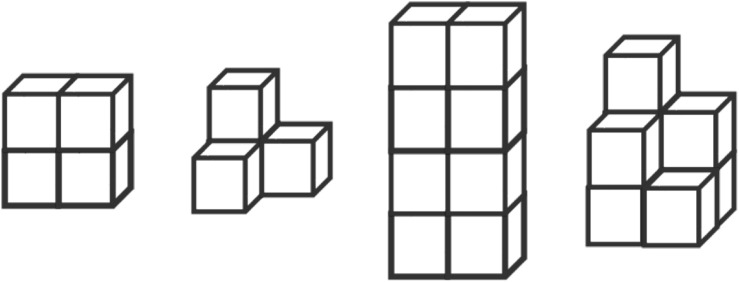
The cube transformation task.

The Counting and Coloring of Solid Cubes Test (CCSCT) was used to assess children’s cube transformation from 2-D to 3-D. Li et al. (1997) developed the CCSCT based on previous research (Moore, 1986). This task included four figures of four or eight stacked cubes, and two of them had hidden cubes (hidden cubes are necessary to build the 3-D construction but hard to see in the 2-D picture) (see Figure 4). First, children were asked to count the number of cubes in the picture by pointing at each of them, and then to paint all the surfaces of the same cube in the same color, and to paint cubes next to each other in different colors. This task was scored from 0 to 48. The original authors did not report the test’s reliability. Cronbach’s α in the current study was 0.84.

The Windows Test (WT) was used to assess children’s 2-D mental rotation (Tzuriel and Egozi, 2010). This test includes three difficulty levels (WT1, WT2, and WT3). Based on the result of a pilot study, second-year preschoolers could not understand and complete WT2 and WT3, so only WT1 was used in this study. The standard figure in this instrument is composed of one triangle roof and nine square windows (three of which are black and six of which are white). Children were asked to find the position of the black windows in the contrast figure which was the original figure rotated 45°, 90°, 180° and with all windows being white. This task was scored from 0 to 18. Cronbach’s α was 0.79 in the original study and 0.84 in the current study.

The total score for spatial visualization was calculated by averaging the standardized scores of cube transformation and mental rotation. Reliability for the total summary scores of overall spatial skills was Cronbach’s α = 0.848.

### Procedure

Children were tested individually in the fall (i.e., the first half of their second year in preschool). One experimenter administered the block-building test and coded the level of block building during the 15-min construction period. The finished product was also photographed, and the experimenter used the photos to verify the original scores after the test. Two research assistants (graduate students), who were not familiar with this study’s goal, tested children’s spatial skills. They strictly followed the instructions of the instruments. The total time for the testing of spatial skills ranged from 30 to 40 min.

#### Power Analysis

Power analyses were conducted using G^∗^power for the two types of main analyses. For bivariate correlations aimed at exploring the associations between spatial skill and block-building ability, the sample sizes needed to yield a power of 0.80 and α = 0.05 (one-tailed, given the known positive correlations between spatial abilities and block-building ability). The score was 67 for *r* = 0.30 (medium effect size) and 23 for *r* = 0.50 (large effect size). For the regression analyses aimed at identifying unique contributors and potential interactive effects with gender, the sample sizes needed to yield a power of 0.80 and α = 0.05 for a hierarchical regression analysis, which was 77 for *f*^2^ = 0.15 (medium effect size) or 36 for *f*^2^ = 0.35 (large effect size) (fixed model, testing R^2^ increase, with two control variables and three predictor variables, assuming there would be three significant correlates based on the bivariate correlations), and 68 for *f*^2^ = 0.15 (medium effect size) or 31 for *f*^2^ = 0.35 (large effect size) (fixed model, testing *R*^2^ increase, with two control variables and two predictor variables). With 66 subjects, the current study was sufficiently powered for the planned bivariate correlations but slightly underpowered for final regression analyses to detect medium or smaller effects.

## Results

### Correlations Between Spatial Skills and Block-Building Ability

Table 1 shows the Pearson correlations among the key study measures. The overall spatial ability was correlated significantly with the block-building ability, *r* (66) = 0.33, *p* < 0.05. Form perception was positively correlated with block-building ability, *r* (66) = 0.41, *p* < 0.01, but spatial visualization was not. Within form perception, significant correlates of the block-building ability included shape naming, *r* (66) = 0.33, *p* < 0.01, shape recognition, *r* (66) = 0.37, *p* < 0.01, and shape composition, *r* (66) = 0.37, *p* < 0.01. The one exception was that solid figure naming had no correlation with block-building ability. Neither of the two measures of spatial visualization (cube transformation and mental rotation) showed significant relation with block-building ability.

**TABLE 1:** Correlations among key study variables.

	M ± SD	1	2	3	4	5	6	7	8	9	10
1. Block building skills	5.45 ± 2.11	–	–	–	–	–	–	–	–	–	–
2. Spatial ability	54.83 ± 18.27	0.33*	–	–	–	–	–	–	–	–	–
3. Form perception	15.98 ± 6.49	0.41**	0.77**	–	–	–	–	–	–	–	–
4. Spatial visualization	38.85 ± 15.44	0.12	0.82**	0.26*	–	–	–	–	–	–	–
5. Shape naming	3.58 ± 1.57	0.33**	0.65**	0.82**	0.24	–	–	–	–	–	–
6. Shape recognition	5.14 ± 1.97	0.37**	0.53**	0.65**	0.22	0.46**	–	–	–	–	–
7. Shape composition	5.86 ± 2.79	0.37**	0.59**	0.80**	0.17	0.57**	0.26*	–	–	–	–
8. Solid figure naming	1.41 ± 2.26	0.15	0.54**	0.74**	0.15	0.42**	0.22	0.58**	–	–	–
9. Cube transformation	27.41 ± 12.33	0.16	0.70**	0.26*	0.82**	0.21	0.18	0.21	0.17	–	–
10. Mental rotation	11.44 ± 5.92	0.04	0.64**	0.17	0.82**	0.18	0.18	0.07	0.08	0.35**	–

### Regression Analysis of Form Perception and Spatial Visualization Predicting Block-Building Ability

Two sets of hierarchical regression were conducted to examine unique predictors of block-building ability. In the first set, we examined whether form perception and spatial visualization (as well as their interactions with gender and age) made unique contributions to block-building ability. Step 1 included two demographic variables: age and gender. Step 2 included two factors: form perception and spatial visualization. Step 3 included the interaction terms one at a time.

**FIGURE 5:**
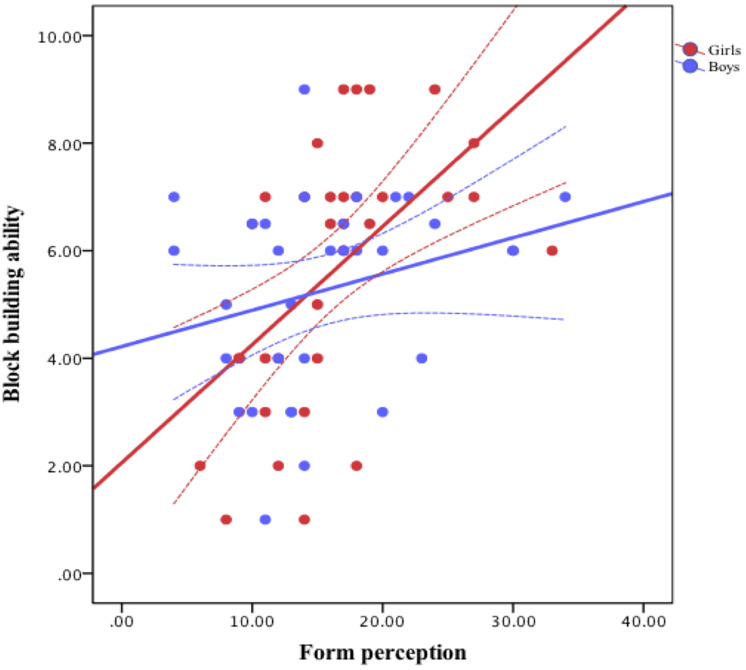
The association between form perception and block building ability by gender.

Results are shown in Table 2. Age was a significant predictor, but gender was not. Form perception made a unique contribution to explaining block-building ability, but spatial visualization did not. These two factors accounted for 9% additional variance. The interaction between form perception and gender was significant. Simple slope tests showed that form perception was significantly associated with block-building ability for girls, with a 1-unit difference in form perception being associated with a 0.73 points difference in block-building ability, *t* = 3.66, *p* < 0.01, but not for boys, *t* = 1.21, *p* = 0.23. Figure 5 depicts the nature of the interaction of form perception and gender. The interaction between spatial visualization and gender was not significant. Nor were the two interaction terms with age.

**TABLE 2:** Hierarchical linear regression of form perception and spatial visualization predicting block building ability (*N* = 66).

	B	SE	*t*	*p*	*F*	*df*	*P*	Adj. *R*^2^	Δ Adj. *R*^2^
Step 1	–	–	–	–	7.03	63	0.002*	0.16	0.16
Age	3.03	0.82	3.68	0.000***	–	–	–	–	–
Gender	–0.27	0.48	–0.55	0.582	–	–	–	–	–
Step 2	–	–	–	−−	6.54	61	0.000***	0.25	0.09
Form perception	0.73	0.24	3.08	0.003**	–	–	–	–	–
Spatial visualization	0.01	0.25	0.04	0.972	–	–	–	–	–
Step 3a (interaction term was added one at a time)	–	–	–	–	6.90	60	0.000***	0.30	0.05
Gender*form perception	1.02	0.47	2.16	0.035*	–	–	–	–	–
Step 3b	–	–	–	–	5.52	60	0.000***	0.26	0.01
Gender*spatial visualization	–0.57	0.50	–1.56	0.252	–	–	–	–	–
Step 3d	–	–	–	–	5.57	60	0.000***	0.26	0.01
Age*form perception	–1.11	0.90	–1.23	0.223	–	–	–	–	–
Step 3e									
Age* spatial visualization	0.27	0.80	0.33	0.742	5.17	60	0.001***	0.24	−0.01

*To simplify the presentation, only new variables for each step are shown because the effects of the variables from earlier steps did not change much in the subsequent steps.*

### Regression Analysis of Specific Spatial Skills Predicting Block-Building Ability

The second set of hierarchical regression was conducted to examine unique predictors (among the specific spatial skills) of block-building ability. Step 1 included two demographic variables: age and gender. Step 2 included the three significant correlates based on the bivariate analyses: shape naming, shape recognition, and shape composition. Step 3 included one of the interaction terms between the three significant correlates and gender or between them and age.

**FIGURE 6:**
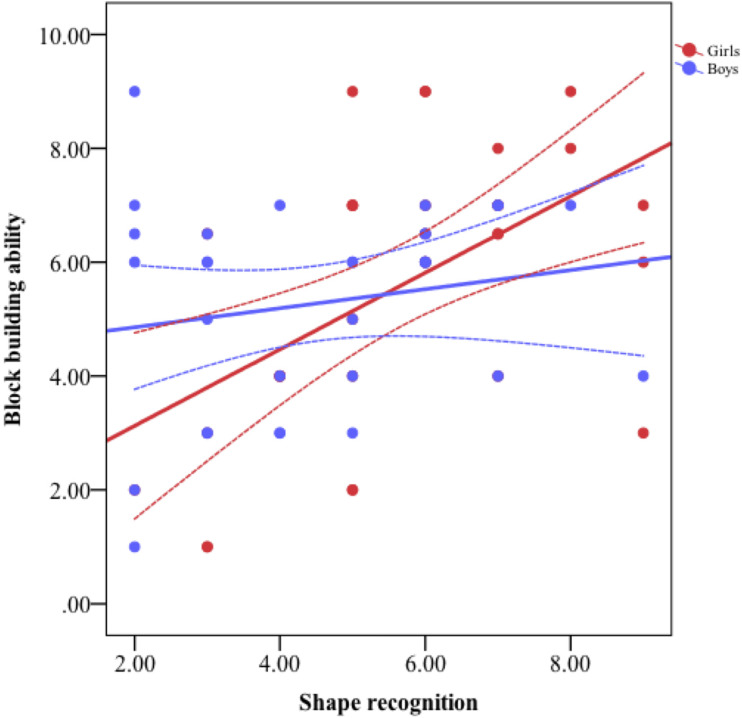
The association between shape recognition and block building ability by gender.

Results are shown in Table 3. Age was a significant predictor, but gender was not. On Step 2, of the three significant correlates based on the bivariate analyses (i.e., shape recognition, shape composition, and shape naming), the former two made unique contributions to explaining block-building ability, but shape naming did not account for a significant amount of unique variance. Step 2 accounted for 13% additional variance. On Step 3, the gender interaction terms of shape naming and shape recognition effects on block building were significant, suggesting that shape naming and recognition’s effects on block-building ability varied by gender. Simple slope tests showed that, although shape naming was associated with block-building ability in the opposite direction (hence a significant interaction), the simple slope was not significant for either girls, *t* = 1.46, *p* = 0.15, or boys, *t* = −1.79, *p* = 0.08. Simple slope tests showed that shape recognition was significantly associated with block-building ability for girls, with a 1-unit difference in shape recognition being associated with a 0.57 points difference in block-building ability, *t* = 3.47, *p* < 0.001, but not for boys, *t* = *−*0.17, *p* = 0.86. Figure 6 depicts the nature of the interaction of shape recognition and gender. None of the interaction terms with age were significant.

**TABLE 3:** Hierarchical linear regression predicting block building ability (*N* = 66).

	*B*	SE	*t*	*p*	*F*	*df*	*P*	Adj. *R*^2^	Δ Adj. *R*^2^
Step 1	–	–	–	–	7.03	63	0.002*	0.16	0.16
Age	3.03	0.82	3.68	0.000***	–	–	–	–	–
Gender	–0.27	0.48	–0.55	0.582	–	–	–	–	–
Step 2	–	–	–	–	6.26	60	0.000***	0.29	0.13
Shape naming	–0.07	0.30	–0.23	0.818	–	–	–	–	–
Shape recognition	0.57	0.26	2.19	0.033*	–	–	–	–	–
Shape composition	0.57	0.27	2.08	0.042*	–	–	–	–	–
Step 3a (interaction term was added one at a time)	–	–	–	–	6.90	59	0.000***	0.35	0.06
Gender*shape naming	–1.16	0.44	–2.64	0.011*	–	–	–	–	–
Step 3b	–	–	–	–	6.91	59	0.000***	0.35	0.06
Gender*shape recognition	–1.19	0.45	–2.65	0.010**	–	–	–	–	–
Step 3c	–	–	–	–	5.61	59	0.000***	0.30	0.01
Gender*shape composition	–0.64	0.46	–1.38	0.174	–	–	–	–	–
Step 3d	–	–	–	–	6.09	59	0.000***	0.32	0.03
Age*shape naming	–1.56	0.80	–1.95	0.056	–	–	–	–	–
Step 3e	–	–	–	–	5.16	59	0.000***	0.28	-0.01
Age*shape recognition	–0.25	0.68	–0.37	0.711	–	–	–	–	–
Step 3e	–	–	–	–	5.33	59	0.000***	0.29	0.00
Age*shape composition	–0.91	1.02	–0.89	0.377	–	–	–	–	–

*To simplify the presentation, only new variables for each step are shown because the effects of the variables from earlier steps did not change much in the subsequent steps.*

## Discussion

The current study aimed at investigating the underlying cognitive mechanism of block-building complexity. We found that three measures of form perception (shape naming, shape recognition, and shape composition) were significantly correlated with block-building complexity, but the fourth measure (3-D shape naming) was not. In addition, neither of the two measures of spatial visualization (mental rotation and cube transformation) were a significant correlate. Finally, form perception and the specific skill of shape recognition had significant interactions with gender.

Our correlation results of form perception and block-building ability shows that block building relies on children’s growing understanding of topological and geometrical knowledge (Hanline et al., 2001) and figure abstraction (Caldera et al., 1999). The more children know about wooden unit blocks containing a variety of shapes (Hsieh and Mccollum, 2018), the more they can manipulate different shapes, and the more complex their patterns of block building become (Stannard et al., 2001). Our quantitative results are consistent with Park et al. (2008) qualitative analysis that found three major actions (i.e., categorizing geometric shapes, composing a larger shape with smaller shapes, and transforming shapes) in free play with wooden unit blocks. The lack of a significant association between 3-D shape naming and block-building complexity was probably due to these young children’s poor understanding of the names of 3-D shapes, with a mean of 1.44, suggesting a floor effect. Children of this age probably use 2-D names to describe 3-D shapes.

Interestingly, there was a gender difference in the association between form perception in general (and shape recognition in particular) and block-building complexity. It seems that girls may benefit more from shape recognition in the development of block-building complexity than boys. Previous research has shown that, compared to boys, girls include more symbolic features of constructions, such as doors and towers (Ramani et al., 2014), and pay more attention to unique shapes (Caldera et al., 1999). Boys seem to be able to build complex constructions regardless of whether they can recognize the shapes correctly. One possible interpretation of this gender interaction is that, compared to boys, girls had significantly higher verbal abilities in childhood (Toivainen et al., 2017) and were more likely to use strategies based on verbalization in the spatial tasks (Tzuriel and Egozi, 2007), so shape recognition (i.e., understanding the labels of shapes) played a more central role in girls’ block building. In contrast, boys were less able to understand the labels (i.e., recognize) shapes, so their block-building ability was hence dependent not on their ability to recognize shapes but simply relied on their shape composition ability as found in this study.

Not surprisingly, we also found that the block construction complexity increased with the chronological age of children, consistent with previous research (Hanline et al., 2001). Interestingly, however, none of the interactions with age were significant. It seemed that within the age range studied, the spatial skills needed for block building were consistent. Future research should expand the age range and investigate the time points at which specific spatial skills may play different roles in block building.

Neither of the two measures of spatial visualization (cube transformation and mental rotation) were significantly correlated with block-building complexity, supporting the limited literature showing a lack of association between spatial visualization and block-building *complexity* in children (Caldera et al., 1999; Casey et al., 2008). Interestingly, a number of previous studies found significant correlations between spatial visualization and block-building *accuracy* (based on structured block-building tasks). It seems that different kinds of block play activities may require different skills and tap into different abilities (Ramani et al., 2014). Free play exposes children to imagination, creativity, problem-solving, and abstract thinking challenges, which can improve the ability of producing complex relations (Verdine et al., 2014b; Otsuka and Jay, 2017), whereas structured block building stimulates spatial visualization, patterning, and transformation (Ramani et al., 2014; Verdine et al., 2014b).

As mentioned earlier, block building has been found to be beneficial for children’s cognitive development and their later school achievement. As a key preschool activity and a reliable measure of children’s intellectual development, block building has gained more and more interest from researchers of early education. Our results provide new insights into the development of children’s block-building complexity. Educators should pay attention to shape knowledge and their differential roles for boys vs. girls to enhance children’s block play complexity.

The present study has several limitations that should be addressed in future research. First, the sample size is small and all participants came from one preschool, which limits the generalizability of our conclusion. Also due to the sample size, we did not further correct for multiple comparisons. Further research with a larger sample is needed to confirm the results. Second, we included only two measures of spatial visualization (2-D mental rotation and 2-D to 3-D transformation) and these tasks appeared to be somewhat difficult for these young children. More measures of spatial visualization with appropriate levels of difficulty for preschoolers are needed before we can firmly conclude that spatial visualization plays little role in block-building ability. Third, we studied only one age group. It is important to examine whether form perception and spatial visualization affect block building differentially at different age levels. Finally, as mentioned above, the spatial abilities related to block-building complexity may be different from those related to block-building accuracy. Therefore, different tasks are needed that can capture both accuracy and complexity in order to compare their differential cognitive mechanisms.

In summary, the current study investigated the relationships between block-building complexity and spatial skills including form perception (shape naming, shape recognition, shape composition, and solid figure naming) and spatial visualization (cube transformation and mental rotation). Form perception measures generally had significant relations with block-building complexity, but those of spatial visualization did not. There was also some evidence that shape recognition (and possibly shape composition) may be more relevant for girls than for boys.

## Data Availability Statement

The raw data supporting the conclusions of this article will be made available by the authors, without undue reservation.

## Ethics Statement

The studies involving human participants were reviewed and approved by Collaborative Innovation Center of Assessment for Basic Education Quality, Beijing Normal University. Written informed consent to participate in this study was provided by the participants’ legal guardian/next of kin.

## Author Contributions

XZ had overall responsibility for the research design, data collection, data analysis, and draft writing. CC was responsible for research design and draft editing, as well as interpreting all the data and results. TY was responsible for analyzing the data. XX was responsible for research design, participants’ employment, and reviewing the manuscript. All authors contributed to the article and approved the submitted version.

## Conflict of Interest

The authors declare that the research was conducted in the absence of any commercial or financial relationships that could be construed as a potential conflict of interest.

## Publisher’s Note

All claims expressed in this article are solely those of the authors and do not necessarily represent those of their affiliated organizations, or those of the publisher, the editors and the reviewers. Any product that may be evaluated in this article, or claim that may be made by its manufacturer, is not guaranteed or endorsed by the publisher.
