# Erratum: Spatial Skills Associated With Block-Building Complexity in Preschoolers

**DOI:** 10.3389/fpsyg.2021.678894

**Published:** 2021-03-30

**Authors:** Frontiers Production Office

**Affiliations:** Frontiers Media SA, Lausanne, Switzerland

**Keywords:** block building complexity, form perception, spatial visualization, spatial skill, preschooler

Due to a production error, there were errors in the affiliations for authors Tao Yang and Xiaohui Xu. For Tao Yang, instead of affiliation 3, it should be affiliation 4, “School of Preschool Education, Capital Normal University, Beijing, China.” For Xiaohui Xu, instead of affiliation 4, it should be affiliation 3, “Department of Psychological Science, University of California, Irvine, Irvine, CA, United States.”

The publisher apologizes for this mistake. The original article has been updated.

