# Corrigendum: Spatial Skills Associated With Block-Building Complexity in Preschoolers

**DOI:** 10.3389/fpsyg.2021.719389

**Published:** 2021-07-21

**Authors:** Xiaoxia Zhang, Chuansheng Chen, Tao Yang, Xiaohui Xu

**Affiliations:** ^1^Collaborative Innovation Center of Assessment for Basic Education Quality, Beijing Normal University, Beijing, China; ^2^School of Education, Capital Normal University, Beijing, China; ^3^Department of Psychological Science, University of California, Irvine, Irvine, CA, United States; ^4^School of Preschool Education, Capital Normal University, Beijing, China

**Keywords:** block building complexity, form perception, spatial visualization, spatial skill, preschooler

In the published article, there was an error regarding the affiliation superscripts for authors Tao Yang and Xiaohui Xu. For Tao Yang, instead of “4,” It should be “1,” and for Xiaohui Xu, instead of “3” it should be “4.”

The authors apologize for these errors and state that they do not change the scientific conclusions of the article in any way. The original article has been updated.

## Publisher's Note

All claims expressed in this article are solely those of the authors and do not necessarily represent those of their affiliated organizations, or those of the publisher, the editors and the reviewers. Any product that may be evaluated in this article, or claim that may be made by its manufacturer, is not guaranteed or endorsed by the publisher.

